# Factitial panniculitis secondary to injected subcutaneous elemental mercury

**DOI:** 10.1016/j.jdcr.2022.01.024

**Published:** 2022-01-31

**Authors:** Sakkaravarthi Vinupriya, K. Gopalakrishnan, Ranjan Shivapriya, P. Sabari Arasu, S. Arun Kumar, Aditya Vijayanarayanan, S. Anand Shankar

**Affiliations:** a Department of Dermatology, KMCH Institute of Health Sciences and Research, Coimbatore, Tamil Nadu, India; b Department of Pathology, KMCH Institute of Health Sciences and Research, Coimbatore, Tamil Nadu, India; c Department of Radiodiagnosis, KMCH Institute of Health Sciences and Research, Coimbatore, Tamil Nadu, India; d Department of General Medicine, KMCH Institute of Health Sciences and Research, Coimbatore, Tamil Nadu, India; e Department of General Surgery, KMCH Institute of Health Sciences and Research, Coimbatore, Tamil Nadu, India

**Keywords:** factitial, injection, mercury, panniculitis, subcutaneous

## Introduction

Factitial panniculitis or artifactual panniculitis is inflammation of subcutaneous adipose tissue secondary to external injury. The injury can be caused by physical, mechanical, or injected chemical agents, which may be deliberate or accidental, or as a result of a iatrogenic intervention.^1^ Deliberate self-injection of elemental mercury is rare and is usually reported in persons with psychiatric illness.^2^ We report a case of factitial panniculitis secondary to injected elemental mercury in an otherwise healthy young adult woman who denied knowledge of the etiology.

## Case report

**Fig 1:**
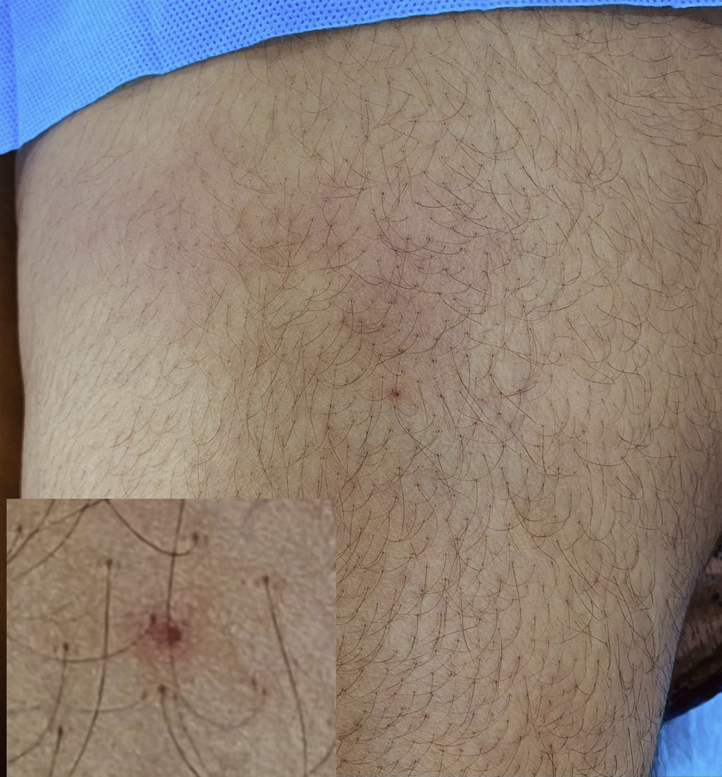
An ill-defined, erythematous, warm, tender, indurated plaque over the left thigh. *Inset*: magnified image of a needle mark.

**Fig 2:**
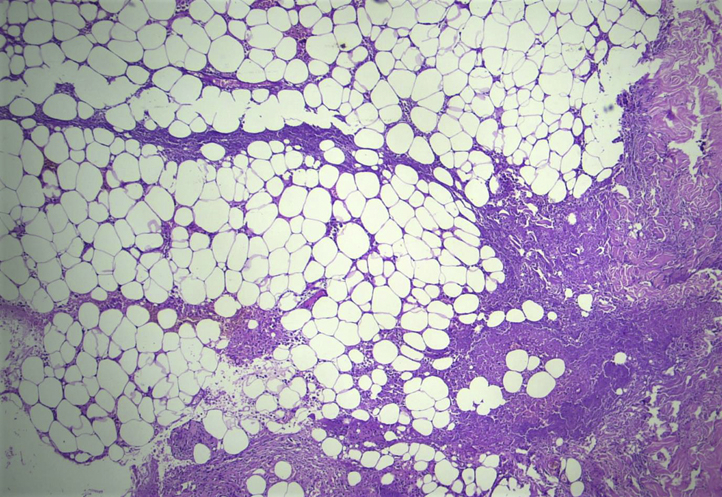
Multifocal fat necrosis with lobular panniculitis and predominantly neutrophilic infiltrates. (Hematoxylin-eosin stain; original magnification: ×10.)

A 21-year-old woman who was working as a cashier in a hospital presented to our outpatient department with sudden onset of painful lesions involving the bilateral arms and thighs for the past 2 days with no history of recent trauma. There were no constitutional symptoms or symptoms suggestive of connective tissue diseases. There were no similar occurrences in the past. The patient had a low-grade fever (38 °C) and multiple ill-defined erythematous, warm, and extremely tender indurated plaques involving the extensor surfaces of the bilateral arms and anterior aspect of the bilateral thighs, with no regional lymphadenopathy (Fig 1). A differential diagnosis of panniculitis secondary to systemic lupus erythematosus/dermatomyositis was made. The laboratory workup revealed neutrophilic leukocytosis and an elevated erythrocyte sedimentation rate. Hepatic and renal parameters, urine routine test, creatine phosphokinase, amylase, lipase, chest roentgenogram, and computed tomography of the chest were all normal. Antinuclear antibody, perinuclear anti-neutrophil cytoplasmic antibody, cytoplasmic anti-neutrophil cytoplasmic antibody, C-reactive protein, and viral markers were negative. The histopathologic examination revealed lobular panniculitis without vasculitis with multiple foci of fat necrosis and dense inflammatory infiltrate predominantly consisting of neutrophils and occasional lymphocytes, foamy macrophages, and giant cells (Fig 2). The patient was started on oral prednisolone (1 mg/kg) and broad-spectrum antibiotics.

**Fig 3:**
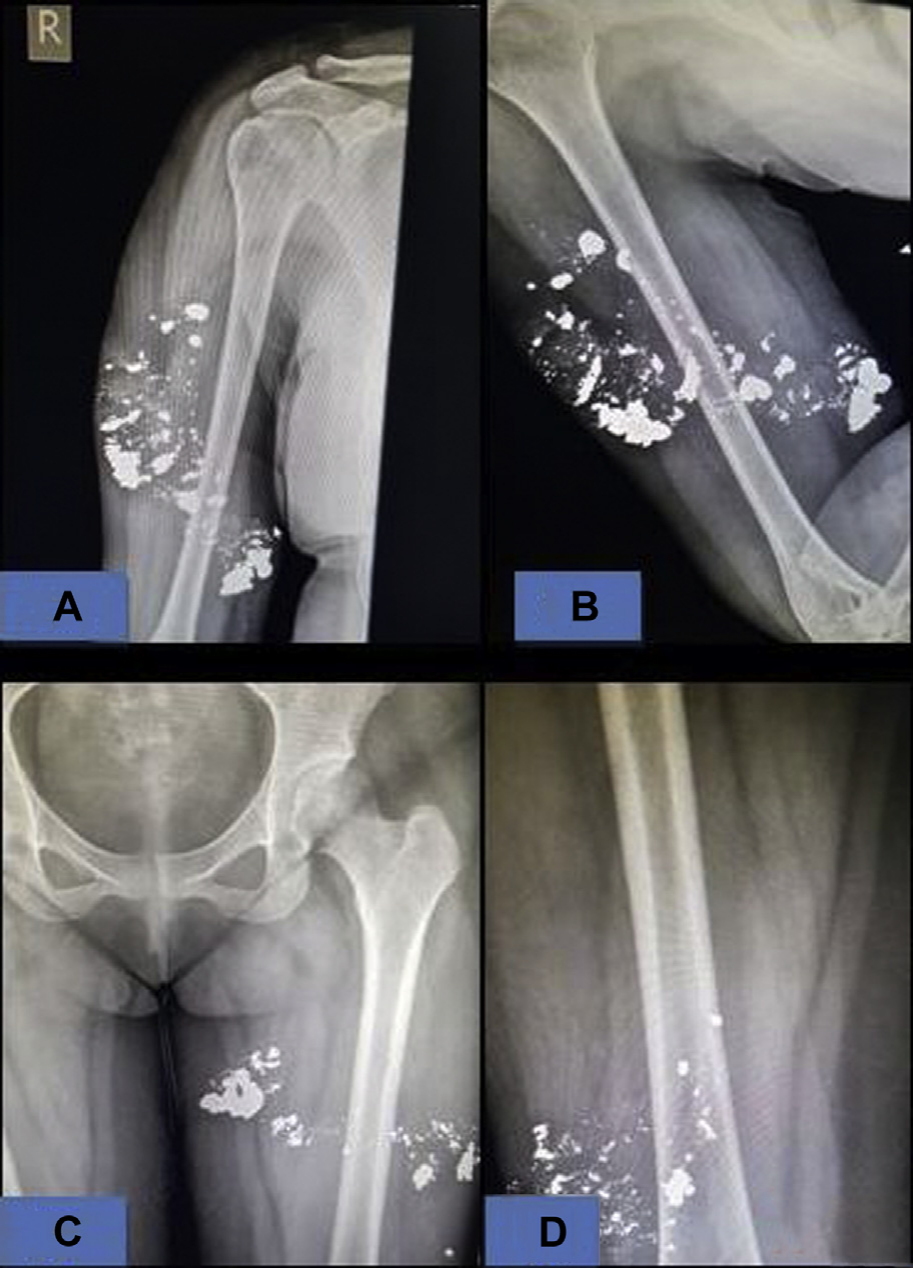
Skiagram showing multiple nodular radiodense opacities in the subcutaneous plane in the right arm **(A)**, left arm posterior view **(B),** left thigh **(C)**, and lower portion of right thigh (**D**).

**Fig 4:**
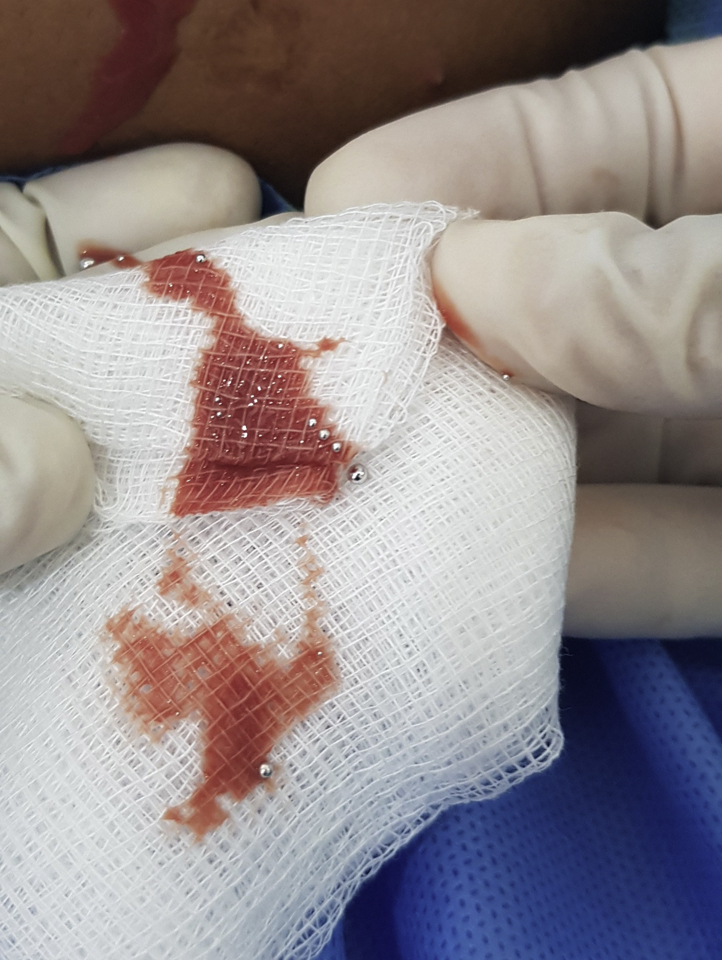
Elemental mercury droplets that emerged with the seropurulent discharge from the left arm.

One week later, she returned with a spontaneous seropurulent discharge from the pre-existing plaques. On examination, all the plaques had become partly fluctuant. Ultrasound examination of all four limbs revealed ill-defined collections with mobile hyperechoic foci in the subcutaneous plane. X-rays revealed multiple dense nodular opacities in the subcutaneous plane of the bilateral arms and thighs (Fig 3). An incision was made to drain the collection over the left arm. To our surprise, we documented the presence of metallic droplets draining along with the thick seropurulent discharge (Fig 4). We carefully collected all the metallic droplets in an unbreakable plastic container with an airtight lid. The sample was sent to the hospital biomedical waste management team, and laboratory analysis confirmed that the metallic droplets were mercury. While reviewing her clinical images at the first visit, we were able to find a needle mark in the plaque over her left thigh (Fig 1, *inset*). The patient was referred to the emergency surgical team for immediate debridement, and psychiatric evaluation was performed with suspicion of deliberate self-injection, but the patient vehemently denied knowledge of the etiology. The patient’s parents were informed about the possibility of self-injection of mercury, and psychiatric counseling was performed.The patient requested discharge from our facility, stating her financial constraints. She was then referred to the state hospital for further treatment.

## Discussion

Factitious disorders are characterized by skin lesions that are self-induced to satisfy an unconscious or conscious psychologic need. This is often done to assume the sick role and not for an external incentive, in which case it would be malingering.^3^ Factitial panniculitis is characterized by inflammation of subcutaneous tissue due to trauma or injection of chemicals or the application of heat or cold. Subcutaneous injection of elemental mercury is rare and is often reported among psychiatric patients.^2^ However, there are a few reports of the use of subcutaneous mercury injections in mentally healthy individuals for various reasons, including self-harm, suicide attempt, weight loss, body building, increase in sexual performance, warding off evil, and accidental exposure,^2^^,^^4^ and in healthy children, to mimic a fictional character.^5^^,^^6^ The most common presentation of subcutaneous elemental mercury injection is painful indurated nodules.^2^^,^^4^^,^^7^ Less common presentations, such as nonhealing ulcer, sterile abscess, and cellulitis-like and morbilliform rash, have also been reported.^5^^,^^6^^,^^8^ Most self-injections are in multiple locations in accessible areas.^2^^,^^4^^,^^5^^,^^6^ To our knowledge, symmetric and panniculitis-like presentation of self-injected mercury has not been previously reported. Histologic examination of subcutaneous mercury is reported to show a granulomatous reaction with mixed inflammatory infiltrates containing neutrophils, lymphocytes, histiocytes, plasma cells, and eosinophils, which was in accordance with our case.^6^

Mercury is a toxic heavy metal that exists in three forms: elemental mercury, inorganic salts, and in organic compounds. Exposure can occur by inhalation, ingestion, or injection.^6^^,^^9^ Systemic toxicity usually affects the neurologic, gastrointestinal, and renal systems. Subcutaneous injection of elemental mercury results in localized abscess formation and usually has no systemic toxicity.^6^ However, mercury levels in the blood and urine can be elevated, mandating chelation therapy with dimercaptosuccinic acid, dimercaprol, or D-penicillamine.^5^, ^6^, ^7^, ^8^ The management of subcutaneous elemental mercury includes debridement of tissues with mercury, monitoring of systemic manifestations of toxicity, chelation therapy in symptomatic cases or cases with elevated blood mercury levels, and psychiatric evaluation of the patient.

Our case shows that injected subcutaneous elemental mercury can present as factitial panniculitis. A symmetric distribution and unforthcoming history can lead to delay in diagnosis. The unregulated sale of mercury via e-commerce websites makes it easily available to the general public for misuse.

## Conflicts of interest

None disclosed.
